# Supplementary material for: Efficacy and Safety of Ticagrelor versus Clopidogrel in Dialysis Patients with Coronary Syndromes: A Systematic Review and Meta-Analysis
Source: J Clin Med. 2023 Jul 30;12(15):5011. doi: 10.3390/jcm12155011 (PMC10419464; doi:10.3390/jcm12155011)
Supplement: Supplementary file 1 [file jcm-12-05011-s001.zip › jcm-2501243-supplementary.pdf]

Table S1. Databases and search strategies used in present systematic review and meta-analysis.

Database	Coverage	Search run	Records
**MEDLINE**	1946 to present	("Dual Anti-Platelet Therapy"[Mesh] OR "Platelet Aggregation Inhibitors"[Mesh] OR antiaggregant OR ticagrelor OR clopidogrel) AND ("Kidney Failure, Chronic"[Mesh] OR "Renal Dialysis"[Mesh] OR end-stage kidney disease OR hemodialysis OR peritoneal dialysis) AND (adverse outcomes OR adverse events OR major adverse cardiovascular events OR mortality OR death OR coronary artery revascularization OR restenosis OR myocardial infarction OR stroke OR target lesion revascularization OR hemorrhage OR bleeding OR major bleeding OR non-major bleeding)	57
		("Dual Anti-Platelet Therapy"[Mesh] OR "Platelet Aggregation Inhibitors"[Mesh] OR antiaggregant OR ticagrelor OR clopidogrel) AND ("Kidney Failure, Chronic"[Mesh] OR "Renal Dialysis"[Mesh] OR end-stage kidney disease OR hemodialysis OR peritoneal dialysis)	64
		(ticagrelor OR clopidogrel) AND ("Kidney Failure, Chronic"[Mesh] OR "Renal Dialysis"[Mesh] OR end-stage kidney disease OR hemodialysis OR peritoneal dialysis)	24
			Total records = 145
**Embase**	1966 to present	('dual antiplatelet therapy'/exp OR 'antithrombocytic agent'/exp OR antiaggregant OR ticagrelor OR clopidogrel) AND ('chronic kidney failure'/exp OR 'hemodialysis'/exp OR end-stage kidney disease OR hemodialysis OR peritoneal dialysis) AND (adverse outcomes OR adverse events OR major adverse cardiovascular events OR mortality OR death OR coronary artery revascularization OR restenosis OR myocardial infarction OR stroke OR target lesion revascularization OR hemorrhage OR bleeding OR major bleeding OR non-major bleeding)	83
		('dual antiplatelet therapy'/exp OR 'antithrombocytic agent'/exp OR antiaggregant OR ticagrelor OR clopidogrel) AND ('chronic kidney failure'/exp OR 'hemodialysis'/exp OR end-stage kidney disease OR hemodialysis OR peritoneal dialysis)	289
		(ticagrelor OR clopidogrel) AND ('chronic kidney failure'/exp OR 'hemodialysis'/exp OR end-stage kidney disease OR hemodialysis OR peritoneal dialysis)	62
			Total records = 434
**Cochrane library**	1967 to present	("Dual Anti-Platelet Therapy"[Mesh] OR "Platelet Aggregation Inhibitors"[Mesh] OR antiaggregant OR ticagrelor OR clopidogrel) AND ("Kidney Failure, Chronic"[Mesh] OR "Renal Dialysis"[Mesh] OR end-stage kidney disease OR hemodialysis OR peritoneal dialysis) AND (adverse outcomes OR adverse events OR major adverse cardiovascular events OR mortality OR death OR coronary artery revascularization OR restenosis OR myocardial infarction OR stroke OR target lesion revascularization OR hemorrhage OR bleeding OR major bleeding OR non-major bleeding)	77
		("Dual Anti-Platelet Therapy"[Mesh] OR "Platelet Aggregation Inhibitors"[Mesh] OR antiaggregant OR ticagrelor OR clopidogrel) AND ("Kidney Failure, Chronic"[Mesh] OR "Renal Dialysis"[Mesh] OR end-	110

		stage kidney disease OR hemodialysis OR peritoneal dialysis)	
		(ticagrelor OR clopidogrel) AND ("Kidney Failure, Chronic"[Mesh] OR "Renal Dialysis"[Mesh] OR end-stage kidney disease OR hemodialysis OR peritoneal dialysis)	75
			Total records = 262
**Scopus**	From the inception till present	("Dual Anti-Platelet Therapy"[Mesh] OR "Platelet Aggregation Inhibitors"[Mesh] OR antiaggregant OR ticagrelor OR clopidogrel) AND ("Kidney Failure, Chronic"[Mesh] OR "Renal Dialysis"[Mesh] OR end-stage kidney disease OR hemodialysis OR peritoneal dialysis) AND (adverse outcomes OR adverse events OR major adverse cardiovascular events OR mortality OR death OR coronary artery revascularization OR restenosis OR myocardial infarction OR stroke OR target lesion revascularization OR hemorrhage OR bleeding OR major bleeding OR non-major bleeding)	0
		("Dual Anti-Platelet Therapy"[Mesh] OR "Platelet Aggregation Inhibitors"[Mesh] OR antiaggregant OR ticagrelor OR clopidogrel) AND ("Kidney Failure, Chronic"[Mesh] OR "Renal Dialysis"[Mesh] OR end-stage kidney disease OR hemodialysis OR peritoneal dialysis)	0
		(ticagrelor OR clopidogrel) AND ("Kidney Failure, Chronic"[Mesh] OR "Renal Dialysis"[Mesh] OR end-stage kidney disease OR hemodialysis OR peritoneal dialysis)	94
			Total records = 94
All databases: 935 records			

Table S2. Quality assessment of included studies using Newcastle-Ottawa scale.

Study	Representativeness of the exposed cohort	Selection of the non-exposed cohort	Ascertainment of exposure	Presence of outcome of interest at the start	Comparability of cohorts	Assessment of outcome	Follow-up long enough for outcomes to occur	Adequacy of follow-up	Total
Jain, 2021	*	*	*		*	*	*	*	7
Lee, 2019	*	*	*		*	*	*		6
Li, 2021	*	*	*	*	*	*	*	*	8
Mavrakanas, 2021	*	*	*		*	*	*	*	7
Tung, 2021	*	*	*	*	*	*	*	*	8

NA = not applicable.
Good quality: 3 or 4 stars in selection domain AND 1 or 2 stars in comparability domain AND 2 or 3 stars in outcome/exposure domain. Fair quality: 2 stars in selection domain AND 1 or 2 stars in comparability domain AND 2 or 3 stars in outcome/exposure domain. Poor quality: 0 or 1 star in selection domain OR 0 stars in comparability domain OR 0 or 1 stars in outcome/exposure domain.
